# A Systematic Literature Review of the Relationship between Serum Ferritin and Outcomes in Myelodysplastic Syndromes

**DOI:** 10.3390/jcm11030895

**Published:** 2022-02-08

**Authors:** Esther Natalie Oliva, Krystal Huey, Sohan Deshpande, Monica Turner, Madhura Chitnis, Emma Schiller, Derek Tang, Aylin Yucel, Christina Hughes, Farrukh Shah

**Affiliations:** 1 Hematology Unit, Grande Ospedale Metropolitano Bianchi Melacrino Morelli, 89124 Reggio Calabria, Italy; 2 Bristol Myers Squibb, Princeton, NJ 08540, USA; krystal.a.huey@gmail.com (K.H.); derek.tang@bms.com (D.T.); Aylin.Yucel@bms.com (A.Y.); Christina.Hughes2@bms.com (C.H.); 3 Evidera, London W6 8BJ, UK; sohan.deshpande@evidera.com; 4 Evidera, Waltham, MA 02451, USA; Monica.Turner@evidera.com (M.T.); madhurakulkarni1082@gmail.com (M.C.); elenoxschill@gmail.com (E.S.); 5 Department of Haematology, Whittington Health NHS Foundation Trust, London N19 5NF, UK; farrukh.shah@nhs.net

**Keywords:** myelodysplastic syndromes, iron overload, serum ferritin, systematic literature review

## Abstract

Anemia is the most common form of cytopenia in patients with myelodysplastic syndromes (MDS), who require chronic red blood cell transfusions and may present high serum ferritin (SF) levels as a result of iron overload. To better understand the potential effects of high SF levels, we conducted a systematic literature review (SLR) to identify evidence on the relationship between SF levels and clinical, economic, or humanistic outcomes in adult patients with MDS. Of 267 references identified, 21 were included. No studies assessing SF levels and their relationship with humanistic or economic outcomes were identified. Increased SF levels were an indicator of worse overall survival and other worsened outcomes; however, the association was not consistently significant. SF levels were a significant prognostic factor for relapse incidence of MDS and showed a significant positive correlation with number of blood units transfused but were not associated with progression to acute myeloid leukemia or the time to transformation. Higher SF levels were also an indicator of a lower likelihood of leukemia-free survival, relapse-free survival, and event-free survival. The SLR suggests that SF levels are associated with clinical outcomes in MDS, with higher levels correlated with number of blood units transfused, frequently indicating worse outcomes.

## 1. Introduction

Myelodysplastic syndromes (MDS) are a heterogeneous group of clonal disorders in hematopoietic stem cells, characterized by ineffective hematopoiesis resulting in abnormally low levels of normal red blood cells (RBCs), white blood cells, platelets, or combinations of these cells [1]. Although patients also suffer from an increased risk of infection or hemorrhage and may even progress to acute myeloid leukemia (AML), anemia is the most common form of cytopenia in MDS [2]. To overcome anemia, patients with MDS require chronic RBC transfusions [3], often resulting in iron overload [4,5], which is reflected by high serum ferritin (SF) levels.

While the gold standard of measurements of iron burden is T2* magnetic resonance imaging (MRI) of the heart and R2* MRI of liver iron concentration (LIC) [6], the SF level marker is an acceptable surrogate marker that is universally available worldwide and especially meaningful [7]. High SF levels indicative of iron overload are generally considered to be >1000 µg/L, which is associated with harmful consequences such as organ damage and increased mortality [8]. The risks of cardiac events and hepatic complications are also increased by iron overload, so this complication of regular transfusions is considered to be an independent prognostic variable of overall survival (OS) [9].

To explore the relationship between SF levels and outcomes, we performed a systematic literature review (SLR) to collect the available evidence on the relationship between SF levels and clinical, economic, and humanistic outcomes in patients with MDS.

## 2. Methods

A systematic search and analysis of the literature was undertaken to identify evidence on the relationship between SF levels and the clinical efficacy, safety, and the economic and humanistic burden of illness in patients with MDS. The SLR was conducted according to the standards set forth by the Preferred Reporting Items for Systematic Reviews and Meta-Analyses (PRISMA) [10,11] and the Cochrane Handbook for Systematic Reviews of Interventions [12].

Searches were developed according to established guidelines [10,11,12,13] to identify studies of interest in Embase and MEDLINE and MEDLINE In-Process (both via Ovid); search strategies included a combination of free-text searches and controlled vocabulary terms (Supplementary Materials Tables S1 and S2). Proceedings from conferences of the American Society of Hematology, the European Hematology Association, and the International Society for Pharmacoeconomics and Outcomes Research were searched for relevant abstracts to identify submissions from 1 January 2018 to 23 April 2020. Bibliographies of systematic reviews and/or meta-analyses reporting SF levels in patients with MDS published since 1 January 2018 were also used to identify additional relevant publications.

Predefined inclusion and exclusion criteria (Table 1) were used to evaluate the titles and abstracts of records identified from the searches, and full-text articles of the abstracts deemed relevant were retrieved and examined. Studies that failed to meet the inclusion criteria or were ineligible for inclusion were rejected, and reasons for rejection were captured. Studies were required to report on the association of SF levels with outcomes of interest in patients with MDS, with the relationship evaluated via univariate or multivariate models. All screening was conducted by two independent investigators; screening decisions required agreement between the two, and any disagreements were resolved by a third investigator. Data extraction was performed by one researcher for studies meeting all inclusion criteria and validated by a second researcher, with discrepancies resolved by a third. Risk of bias in the included studies was assessed via the Quality in Prognostic Studies (QUIPS) tool [14].

## 3. Results

**Figure 1:**
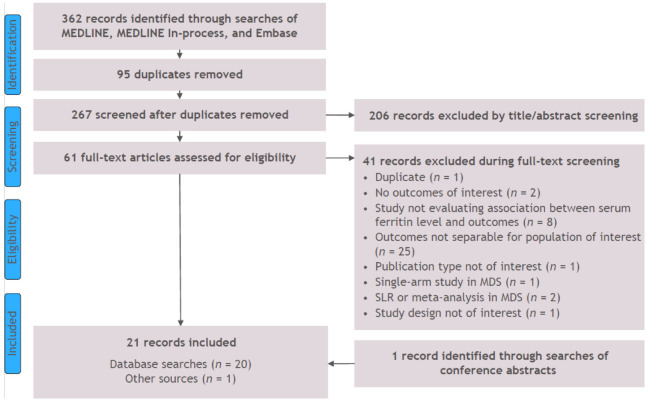
PRISMA diagram of study attrition for MDS studies. Abbreviations: MDS, myelodysplastic syndromes; PRISMA, Preferred Reporting Items for Systematic Reviews and Meta-Analyses; SLR, systematic literature review.

Searches were conducted on 23 April 2020, returning 362 references. From these, 95 duplicates were removed, and 267 abstracts were screened. Among them, 61 references were evaluated at the full-text level, 41 of which were excluded. Supplementary searches of conference presentations identified an additional reference, resulting in 21 studies eligible for inclusion in the SLR (Figure 1) [15,16,17,18,19,20,21,22,23,24,25,26,27,28,29,30,31,32,33,34,35].

Risk of bias assessed via the QUIPS tool indicated that studies were generally of good quality with low risk of bias. Baseline characteristics were inconsistently reported across five studies [15,16,21,25,34], and three studies were unclear in their information on variables in multivariate models [19,21,30]. Despite these minor gaps in information, studies consistently reported other elements (study attrition, prognostic factor, outcome measurement, and statistical discussion), resulting in a low risk of bias across the remaining categories (Supplementary Materials Table S3).

Of the 21 observational studies identified [15,16,17,18,19,20,21,22,23,24,25,26,27,28,29,30,31,32,33,34,35], most (*n* = 15) examined retrospective cohorts, while six reported on prospective cohorts. Geographic locations across studies varied: three studies in Japan [22,23,27]; two each in the United States [26,29], Canada [30,35], the Czech Republic [16,21], and Turkey [15,32]; and one each from China [24] and New Zealand [20]. The remaining data were from a range of European countries. When the study setting was reported, data were measured in hospital settings, outpatient settings, cancer centers, and hematology-specific sites and centers. Most studies (*n* = 17) evaluated at least 50 patients, although the study populations ranged from 35 patients in two studies [15,19] to 419 in a cohort of patients treated at four hematologic centers in Austria (Table 2) [33].

Study population mean ages ranged from 49.4 to 69.8 years, and median ages from 50 to 77 years (Table 2). Most studies included between 47% and 70.6% male participants, though one subgroup of 10 patients with high SF levels included 90% male participants. None of the studies included information on race, and only one study presented information on the ethnicity of the patient population [20]. Time since diagnosis or time between diagnosis and treatment were infrequently reported and ranged from a median of 8.0 to 16.5 months in the three studies reporting those data [17,26,31]. Only nine studies reported information on transfusion dependence or whether patients required transfusions [15,18,21,23,28,29,30,31,34]. Patient populations and inclusion criteria varied; values ranged from 0% of patients who were transfusion dependent in a French registry study in non-transfusion dependent patients [28] to 69% of patients requiring a transfusion in a retrospective cohort study [29]. SF levels at baseline varied within and across studies.

Disease status was reported using several indicators: International Prognostic Scoring System (IPSS) and revised International Prognostic Scoring System (IPSS-R) risk groups, as well as French–American–British (FAB) and World Health Organization (WHO) risk groups. The included studies reported both univariate and multivariate models, with six studies using both to analyze the association between SF levels and outcomes. Studies used a variety of statistical approaches to assess the prognostic value of SF levels, including Cox proportional hazards, Pearson product–moment correlation coefficient or Spearman’s rho test, Wilcoxon’s test, Mann–Whitney U test, Chi-squared tests, and regression analyses. Variables for which the models controlled were inconsistently reported. When reported, these varied across studies and included age, sex, IPSS subgroup, disease stage or progression, and bone marrow (BM) blasts. A variety of outcomes were predicted using these models. Outcomes were limited to clinical outcomes; no data were identified on economic or humanistic outcomes associated with SF levels.

### 3.1. Survival and Mortality

Across the identified literature, most studies (*n* = 16) explored the association between SF levels and survival or mortality outcomes; the outcomes evaluated included OS, worsened survival, non-relapse mortality (NRM), and transplantation-related mortality [16,17,18,21,22,23,24,26,27,28,29,30,32,33,34,35]. Results are provided in Supplementary Materials (Table S4).

Findings from the included studies suggested that higher SF levels were associated with reduced survival, although it was difficult to identify strong patterns across the 16 studies. Eleven studies demonstrated the prognostic value of SF levels in OS, with higher SF often indicating worse OS [16,17,18,21,22,23,24,26,27,30,33]. Patients with SF levels >210 ng/mL had significantly poorer survival compared with that of patients with SF levels above that limit (hazard ratio [HR]: 2.14; 95% confidence interval [CI]: 1.02–4.50; *p* = 0.044) [22]. Higher SF levels measured on a continuous scale were a significant predictor of worse OS in a study of a cohort of 419 patients with primary MDS who were treated at one of four Austrian hematologic centers [33]. For the overall cohort, a log scale increase in SF levels was associated with worse OS (HR: 2.2; *p* < 0.01). Among a subgroup of patients, those with Low- or Intermediate (Int)-1-risk MDS experienced a greater reduction in OS (HR: 2.5; *p* < 0.01). The authors noted that during the other analyses conducted in the study, the hematopoietic stem cell transplant (HSCT)-specific comorbidity index and the Charlson Comorbidity Index were independent predictors of worse OS in the overall cohort. However, when SF levels were incorporated into the model, neither remained an independent predictor of OS. The authors suggested that SF levels may lead to organopathy and comorbidities, further complicating the assessment of SF level as a prognostic factor [33].

Five studies found no association between higher SF levels and reduced OS [16,28,29,33,35]; however, when examining specific patient subgroups, two studies did find such a relationship [16,33]. SF levels <2000 µg/L were associated with better survival outcomes among non-transplanted patients, but not among the overall study population [16]. A second study of a French registry, evaluating patients with lower-risk, non-transfusion-dependent disease, found no relationship between SF level and OS when SF levels were >300 ng/mL or >1000 ng/mL (*p* = 0.98 and *p* = 0.67, respectively, in univariate analyses). The study reported that the five-year OS for patients with SF levels < 300 ng/mL was 62% compared with 64% in patients >300 ng/mL. Similarly, in patients with SF > 1000 ng/mL, the five-year OS was 67% compared with 62% in patients with levels < 1000 ng/mL [28]. The studies used various thresholds to define when SF levels were elevated. In one study, patients with SF level > 400 ng/mL had a median OS of 24.2 months compared with a median 42.6 months in patients with SF levels < 400 ng/mL (*p* = 0.003) [18]; a separate study also used this threshold to define elevated SF levels and found similar significant results (mean survival for SF level < 400 ng/mL: 77.2 months; mean survival for SF level ≥ 400 ng/mL: 44.2 months; *p* = 0.001) [32]. In a Japanese study of patients with MDS who did not receive iron chelation therapy (ICT), 5-Aza-2′-deoxycytidine (5-AZA), or HSCT, patients with SF levels ≥500 ng/mL had an HR of 10.7 (95% CI: 2.375–48.23; *p* = 0.002) and an OS of 10.2 months, compared with 118.8 months for patients with SF levels < 500 ng/mL (*p* = 0.001) [23]. A retrospective review of registry data from Poland evaluated the relationship of SF with multiple outcomes, including worsened survival [34]. In the univariate analysis, the study reported an increased risk of worsened survival for patients with SF levels > 1000 ng/mL, with a significantly increased chance of experiencing the outcome (HR: 2.94; *p* = 0.0023).

Though several studies included patients who underwent HSCT, only two assessed transplantation/treatment-related mortality (TRM) or NRM, defined as death from causes other than a relapse of MDS [17,26]. The first study analyzed a US-based cohort and sought to determine whether the disease characteristics at diagnosis of MDS and at the time of HSCT affected patient outcomes; in the cohort, more than 40% were in the High- or Very High-risk IPSS-R category. In the univariate analysis, SF levels >1130 µg/L at the time of HSCT indicated a significantly increased risk of transplantation-related mortality (HR: 2.0; *p* = 0.009). Upon multivariate analysis (controlling for age, donor type, conditioning intensity, and transplantation year), the association was no longer significant, although the trend was similar (HR: 1.7; *p* = 0.06) [26]. A second study that evaluated patients with MDS who underwent HSCT examined TRM. While this was not defined explicitly, it was evaluated as being the same as “transplantation-related mortality.” Among patients with MDS who underwent HSCT, continuous SF in units of 1000 ng/mL was assessed as a potential prognostic factor for NRM; in the multivariate analysis (controlling for age, sex, transfusions, comorbidities, C-reactive protein levels, WHO classification, and conditioning regimen at HSCT), there was no significant relationship between increase in SF levels and the risk of NRM (HR: 1.1; 95% CI: 0.8–1.4; *p* = 0.06) [17].

### 3.2. Progressive Disease and Relapse

Across the eight studies identified by the SLR that reported on progressive disease (PD) or relapse-related outcomes [17,21,23,26,28,30,33,34], there was a general trend for higher SF levels to predict worse outcomes. However, there was limited evidence on each of these outcomes; three studies reported event-free survival (EFS) [21,26,33], two studies relapse-free survival (RFS) [17,30], two studies relapse incidence [17,26], one study leukemia-free survival (LFS) [23], and two studies transformation to AML/time to transformation [28,34]. Multivariate models controlled for different covariates of interest across studies, including age and sex, as well as histological subtype, BM blast counts, karyotypes, and conditioning regimen at the time of HSCT; these variables were not reported for all studies. Results are shown in the Supplementary Materials (Table S5).

#### 3.2.1. Event-Free Survival

In the three studies reporting EFS, an event was defined as PD or death. Definition of PD included transformation to AML, an increased number of BM blasts, a higher degree of cytopenia, or advancing in FAB classification. Overall, these studies suggested that higher SF levels indicated worse EFS, but the association between SF level and EFS was not demonstrated consistently. Specifically, higher SF levels were significantly associated with worse EFS in two studies using multivariate models, with SF analyzed in a continuous manner (HR: 1.14; *p* = 0.001 [21] and HR: 2.00; *p* < 0.01 [33]). The same trend was demonstrated in a subgroup of Low- and Int-1-risk patients through a multivariate model (HR: 2.9; *p* < 0.01) [33]; however, in a subgroup of Int-2- or High-risk patients in the same study, higher SF levels were not significantly associated with worse EFS, and the trend was much less pronounced (HR: 1.2; *p* = not reported [33]). A third study evaluating patients who underwent HSCT found that SF levels at the time of transplant were significantly associated with worse EFS in univariate and multivariate models [26]. When patients had an SF threshold >1130 µg/L, EFS was significantly worse (HR: 1.6; *p* = 0.01). When this threshold was increased to 1150 µg/L, the HR increased as well (HR: 1.8; *p* = 0.002). The study also compared patients with missing SF levels to those with SF ≤1130 µg/L (univariate; HR: 1.5; *p* = 0.05) and SF ≤1150 µg/L (multivariate; HR: 1; *p* = 0.9); none of these comparisons indicated a significant relationship between SF and EFS, though missing SF level data are likely not an informative subgroup for general comparison [26].

#### 3.2.2. Relapse-Free Survival

In general, lower SF was numerically associated with better RFS in the two studies reporting on this outcome, but the association was not always significant. The two studies evaluated patients who underwent HSCT and reported on the association between pre-transplant SF levels and RFS, and neither study provided a clear definition of “relapse” [17,30]. This relationship was not significant in a multivariate model assessing pre-transplant SF as a continuous measure in units of 1000 ng/mL (HR: 1.2; 95% CI: 0.98–1.4; *p* = 0.08) [17]. However, in the study comparing pretransplant SF levels ≤1000 ng/mL to >1000 ng/mL, the univariate and multivariate analyses found that a lower SF level (below the threshold) was significantly associated with better RFS (HR: 1.931; 95% CI: 1.239–30.10; *p* = 0.0037 in univariate analyses and HR: 1.799; 95% CI: 1.147–2.823; *p* = 0.0106 in multivariate analyses) [30].

#### 3.2.3. Relapse Incidence

The two studies reporting on relapse incidence offered conflicting results. The first study, a US-based retrospective analysis of a cohort of patients who underwent HSCT, defined relapse as “a hematologic recurrence of MDS according to standardized criteria”; SF was not a predictor of relapse incidence in the univariate analysis. When using SF ≤1130 µg/L as a reference, patients with SF levels above that threshold had an HR of 1.0 (*p* = 0.8), while patients with missing SF data showed a slight trend towards greater relapse, although not significant (HR: 1.7; *p* = 0.06) [26]. In the second study, a prospective analysis of patients who underwent HSCT, the multivariate analysis of SF in a continuous manner (in units of 1000 ng/mL) indicated that pre-transplant SF levels had a small but significant association with incidence of relapse after HSCT (HR: 1.3; 95% CI: 1.01–1.6; *p* = 0.04) [17]. Relapse was not defined in that study.

#### 3.2.4. Leukemia-Free Survival

SF level was associated with worse likelihood of LFS in a retrospective analysis of a cohort of patients from Japan who did not receive ICT, 5-AZA administration, or a stem cell transplant [23]. The univariate logistic regression analysis suggested that SF levels ≥500 ng/mL were significantly associated with poor LFS, albeit with a wide confidence interval (HR: 21.16; 95% CI: 2.062–217.1; *p* = 0.01). However, the significance of the association was not maintained when the threshold was set at ≥300 ng/mL (HR: 4.752; 95% CI: 0.852–26.51; *p* = 0.076).

#### 3.2.5. Transformation to AML

Two studies evaluating the relationship between SF and progression to AML, or time to progression to AML, could not find a statistically significant association [28,34]. Both studies presented results from univariate models. While neither study reported an effect size, they both found that there was no association with SF levels >1000 ng/mL and transformation to AML (*p* = 0.47 [28] and *p* > 0.05 [34]), as well as with a lower threshold of SF >300 ng/mL (*p* = 0.94) [28]. SF levels >1000 ng/mL were not shown to be associated with time to transformation to AML when the outcome was evaluated in a cohort from the MDS-Polish Adult Leukemia Group (PALG) registry (*p* = 0.35) [34].

### 3.3. Additional Outcomes

Five studies reported on treatment response, medication adherence, blood units transfused, and liver stiffness measurements (Supplementary Materials, Table S6) [15,19,20,25,31]. A retrospective cohort study in Turkey suggested that lower SF levels at the time of MDS diagnosis were significantly associated with better treatment response (*p* = 0.004). Treatments received by patients within this study were antithymocyte globulin and prednisolone, thalidomide and or lenalidomide, 5-AZA, or thalidomide and 5-AZA [15]. When adherence was evaluated, deferasirox-adherent patients had statistically significantly lower SF compared with that of nonadherent patients (*r* = −0.288; *p* = 0.004) [19]. Two studies noted a positive correlation between SF levels and the number of blood units received, with increases in SF correlated with increases in blood units [20,25], but only one reported it as significant (coefficient: 0.52; *p* = 0.04) [25]. Finally, in a study evaluating a potential tool to assess liver fibrosis in MDS, the univariate analysis indicated that there was no association between higher SF levels (defined as >320 μg/L in men and >161 μg/L in women) and higher liver stiffness measurements (*p* = 0.583) [31].

## 4. Discussion

This SLR on the association between SF levels and outcomes of interest in patients with MDS identified a breadth of clinical findings from a variety of research settings, although none of the included studies presented relevant results on the impact on humanistic or economic outcomes. These studies suggested that SF levels can serve as a prognostic factor; however, the variation in SF level thresholds used when measuring the same outcome, descriptions of what dictates “higher” SF levels, and the lack of consistently significant results (often for the same outcome, at times within the same study) highlight the difficulty in its broad application. Studies overwhelmingly reported on survival-related outcomes, and the results generally suggested that increased SF levels were associated with worse survival outcomes (whether for OS, EFS, or RFS). Despite this, there was no obvious association between SF levels and the incidence of relapse or PD. This may be due in part to the small body of evidence identified; while a large proportion of studies reported that SF levels were associated with worse OS, few studies reported on EFS or RFS, limiting the opportunity to identify trends across these outcomes. Contrasting results, across and within studies, complicated the comparisons, likely due to differing model variables.

Subgroup comparisons, where reported, also provided challenges resulting from the nature of MDS. For example, conflicting EFS results between a subgroup of Low- and Int-1-risk patients and a subgroup of Int-2- and High-risk patients underscored the potential interaction between SF and comorbidities or risk classification, even when such variables were controlled for. At the same time, however, results illustrated that SF was associated with worse survival outcomes in patients with Low- and Int-1-risk classification, suggesting that the measure retains value as a prognostic tool in these patients.

Generally, there was a low risk of bias observed across the studies included in the SLR. While the review itself was subject to the same limitations as all SLRs (namely, publication bias), its reproducible study design and limited opportunity for bias, demonstrates a high degree of rigor. The findings of this review were also limited by the number of included studies reporting univariate analyses only, and very few outcomes had findings confirmed across univariate and multivariate analyses. The results were also somewhat limited due to fact that no data on economic or humanistic outcomes were identified. Future research is needed to fill this data gap to properly explore the relationship between economic or humanistic outcomes and SF levels. Given the impact that increased SF levels has on survival, it would be important to understand the ways in which SF levels impact other facets of the lives of patients with MDS.

With chelation necessary to help address high SF levels and the physical burden of iron overload, a separate SLR was undertaken to review SLRs reporting on the burden of ICT in patients with MDS. Few data were systematically identified, and those that were identified focused primarily on the survival benefits conferred by ICT. Subsequent targeted searches were undertaken to fill data gaps, with evidence suggesting that the use of ICT was costly to patients but did not provide a significant added benefit in quality of life. Patients with MDS receiving ICT had demonstrably worse quality of life compared with that reported for the general population [36]. Similarly, treatment with ICT over time did not provide meaningful improvement in quality of life [37]. In addition, patients experienced a substantial cost burden resulting from their ICT treatment, with 10-year systems-level costs reaching as high as USD 2 million with deferasirox [38]. The evidence in that companion review suggests that where high SF levels require ICT, clinical, humanistic, and economic outcomes are affected. Additional studies exploring the relationship between SF levels and outcomes would likely inform the burden of ICT use, as well.

## 5. Conclusions

The range of evidence identified by this SLR suggests that higher levels of SF are potentially a prognostic indicator for decreased survival in patients with MDS. Additional research using consistent study methods that reduce variation in how results are presented would allow for improved comparisons across studies and populations. Further evidence is needed to determine whether SF levels are associated with economic or humanistic outcomes in these patients.

## Figures and Tables

**Table 1:** Screening/eligibility criteria for myelodysplastic syndromes (MDS).

Domain	Inclusion Criteria	Exclusion Criteria
Population	Adult (≥18 years) patients with MDS	Publications reporting on patient populations in the following categories: Children Healthy volunteers
Prognostic/predictive factors	Studies must have assessed and reported SF levels using quantitative methods *	NA
Outcomes	Clinical outcomes Incidence of complications related to iron overload, including cardiac failure, hypogonadism, hypothyroidism, carcinoma, diabetes, liver failure Time to development of AML Progression to high-risk disease PFS OS Treatment duration Subsequent therapies, or combinations of different types of ICTs, or maintenance on personalized regimen Total mortality Liver fibrosis, stiffness, or siderosis Skeletal outcomes such as bone disease, density, osteoporosis, skeletal changes, or fracture Cardiac siderosis Pulmonary hypertension Fertility Humanistic outcomes Utility studies HRQoL (e.g., EQ-5D, SF-36, and EORTC QLQ-C30) Economic outcomes: Healthcare resource utilization ○ Specialist visits ○ Unscheduled physician visits ○ Emergency room visits ○ Transfusion clinic visits ○ Hospitalization Costs ○ Direct ○ Total treatment ○ Healthcare and social care ○ Indirect ○ Productivity ○ Absenteeism and presenteeism	Publications that only report data on the following types of outcomes: pharmacokinetics/pharmacodynamics
Study designs	Observational cohort studies (prospective or retrospective) RCTs	Publications of studies with the following designs: Animal studies In vitro/ex vivo studies Gene expression/protein expression studies Narrative publications Nonsystematic reviews Case studies Case reports Editorials
		Systematic reviews and meta-analysis (will be included for reference checking only. Full papers will be excluded using a separate exclusion code)
Duplicate	If duplicates are identified, the copy of the record with the lower ref ID number will be included	Publications that are duplicates of other publications in the search yield The copy of the record with higher ref ID number will be excluded
Study limits	Only English language articles/conference abstracts were included	Journal articles and conference abstracts not in the English language
	Studies published from 2009 to present † Conference proceedings from 2018 to present were searched	Studies published outside the timeframe of interest
Geography	None	

* Studies assessing efficacy of an intervention without investigating relationship of SF level and outcomes were excluded. Studies assessing relationship between SF level and any of outcomes of interest listed in table above are of interest. Studies with quantitative outcomes refer to those based on univariate or multivariate models or adjusted analysis with some kind of quantification results, as opposed to studies making unsupported statements about association in discussion (which would be excluded). † This limit ensured we had most recent and relevant data as older studies did not account for impact of current disease management on association between SF levels and outcomes. Abbreviations: AML, acute myeloid leukemia; EORTC QLQ-C30, European Organisation for Research and Treatment of Cancer Quality of Life Questionnaire Version 3.0; EQ-5D, EuroQoL 5-dimension health survey; HRQoL, health-related quality of life; ICT, iron chelation therapy; MDS, myelodysplastic syndromes; NA, not applicable; OS, overall survival; PFS, progression-free survival; RCT, randomized controlled trial; SF, serum ferritin; SF-36, 6-item Short Form Survey.

**Table 2:** Patient characteristics of studies.

Study Author, Year, Country	Study Design, Setting, Sample Size	Age	Male Gender	Duration Since Diagnosis	Proportion Transfusion Dependent and Definition	IPSS Risk Groups	FAB Risk Groups	WHO Risk Groups	Mean SF at Baseline *
Irwin, 2011 [20] New Zealand	Retrospective cohort Hospital 70	Mean (SD), years: 69.8 (NR)	60%	NR	NR defined as the requirement of at least 1 RBC unit per 8 weeks, over at least 4 months	Low: 41.5% Int-1: 35.0% Int-2: 15.0% High: 8.3%	Not diagnostic but consistent with MDS: 11.4% RA: 37.0% RARS: 12.8% RAEB: 28.6% RAEB-t: 0% CMML: 10.0%	Not diagnostic but consistent with MDS: 7.8% del(5q): 4.7% RA: 12.5% RARS: 12.5% RCMD: 25.0% RCMD-RS: 1.6% RAEB-1: 20.3% RAEB-2: 10.3% MU: 4.4%	2963 µg/L
Park, 2011 [28] France	Registry NR/unclear 318	Median (range), years: 77 (29–103)	56%	NR	0 (at registry entry)	0: 44% 0.5: 25% 1: 11% NA by ≤1: 20%	NR	RA: 21% RCMD: 18% RARS and RCMD-RS: 25% RAEB-1: 20% del(5q): 5% Unclassifiable: 14%	Median (range), ng/mL: 283 (20–5558)
Waszczuk-Gajda, 2016 [34] Poland	Retrospective cohort In- or outpatient hematologic unit 190	<50 years: 8%;50–80 years: 77%; >80 years: 15%	58%	NR	58 Defined as having at least 1 RBC transfusion within the last 8 weeks over a period of 4 months	(Available for a subset of 62 patients) Low risk: 16% Int-1: 34% Int-2: 29% High risk: 21%	NR	RA: 12.6% RARS: 3.7% RCMD: 26.3% RCMD-RS: 0.5% RAEB-1: 14.2% RAEB-2: 27.9% del(5q): 2.1% MDS-U: 4.2%	≤1000 µg/L: 89 patients (81.7%) >1000 µg/L: 20 patients (18.3%)
Cakar, 2013 [15] Turkey	Retrospective cohort Blood center records 35	Median (IQR), years: 60 (22–84)	60%	NR	62.8% needed a transfusion during follow-up	(Available for a subset of 33 patients) Low: 30.3% Int-1: 60.6% Int-2: 9.1%	NR	RCUD: 48.6% RCMD: 2.9% RAEB-1: 28.6% RAEB-2: 17.1% Isolated del(5q): 2.9%	At diagnosis: median (range), ng/mL: 198 (6.6–794)
Cermak, 2009 [16] Czech Republic	Retrospective cohort NR/unclear 137	Mean (SD), years: 49.4 (17.8)	54%	NR	NR	Low: 21.2% Int-1 and Int-2: 78.8%	NR	RCMD, RCMD-RS: 64.2% RA, RARS, del(5q): 35.8%	>2000 µg/L: 68 patients (49.6%)
Cremers, 2019 [17] European multi-country	Prospective cohort Hospital 222	Median (range), years: 59.3 (19–76)	NR	Median interval between diagnosis and HSCT: 10 months (range 1–128)	NR	NR	NR	RA/RAS/del(5q)/RCMD-RS: 26% RAEB-1/RAEB-2: 56% AML-MDS: 7% CMML: 11%	Median (1st–3rd Quartile): 700 (261–1554) ≤1000 ng/mL: 115 patients (58%) >1000 ng/mL: 81 patients (41%)
Diamantopoulos, 2019 [18] Greece	Retrospective cohort NR/unclear 88	Median (range), years: 73.4 (35–89)	70.6%	NR	RBC transfusion needed: 46.6%	Low: 9% Int-1: 53.8% Int-2: 37.2% High: 0%	NR	CMML-1: 65.9% CMML-2: 34.1%	Median (range), ng/dL: 333 (24–1541)
Escudero-Vilaplana, 2015 [19] Spain	Retrospective cohort Hospital 35	Median age at beginning of deferasirox treatment: 68.0 years	51.4%	NR	NR	NR	NR	NR	Median (p25–p75), µg/L: 1636 µg/L (1100–1834)
Kadlckova, 2017 [21] Czech Republic	Prospective cohort Outpatient or clinic 73	NR	47%	NR	50.1%	NR	NR	RA, RCUD, RARS, RCMD, MDS-U and MDS with isolated del(5q): 68.5% RAEB-1/RAEB-2: 23.3% CMML: 8.2%	NR
Kawabata, 2019 [22] Japan	Prospective cohort NR/unclear 107	Median (range), years: 70 (20–94)	67.3%	NR	NR	NR	RA: 79.4% RARS: 17.8% CMML: 2.8%	MDS-isolated-del(5q): 1.9% MDS-SLD: 21.5% MDS-RS-SLD: 1.9% MDS-MLD: 42.1% MDS-RS-MLD: 14% MDS-F: 0.9% MDS-U: 11.2% CMML-0: 2.8% MDS/MPN-RS-T: 1.9% MDS/MPN-U: 1.9%	Median (range), ng/mL: 204 (<7 to 14,040)
Kikuchi, 2012 [23] Japan	Retrospective cohort Hospital 47	Low SF group, median (range), years: 67 (27–86); High SF group, median (range), years: 63 (39–74)	Low SF group (*n* = 37): 51.4%; High SF group (*n* = 10): 90%	NR	0	Low SF group (*n* = 37): Low: 18.9% Int-1: 56.8% Int-2: 18.9% High: 5.4%; High SF group (*n* = 10): Low: 0% Int-1: 40% Int-2: 50% High: 10%	NR	Overall: RCUD: 34% RCMD: 36.2% RA + RAEB-1: 19.1% RA + RAEB-2: 10.6%	Low SF group (*n* = 37): Median (range), ng/mL: 158.7 (4.0–475.6); High SF group (*n* = 10): Median (range), ng/mL: 793.5 (519.0–1844.0)
Li, 2013 [24] China	Prospective cohort Hospital 191	Median (range), years: 50 (12–83)	62%	NR	NR	Int-1: 100%	NR	RA: 9% RARS: 9% RCMD: 58% RAEB-t: 15% del(5q): 0.5% MDS-U: 8%	Median (range), µg/L: 368 (8–3256)
Lucijanic, 2016 [25] Croatia	Prospective cohort NR/unclear 36	Median (range), years: 74 (53–89)	53%	NR	NR	NR	NR	RA: 36% RARS: 33% RAEB: 19% RAEB-1: 2/36 (6%) RAEB-2: 5/36 (14%) MDS-U: 8% del(5q): 1/36 (3%)	Unclear timepoint; median (range), µg/L: *HFE* mutated patients: 1113; *HFE* wild-type patients: 458
Oran, 2014 [26] USA	Retrospective cohort Cancer center 256	Median (IQR), years: 56 (48–62)	NR	Median (IQR), months): 8 (5.2–15.3)	NR	IPSS-R at diagnosis: Very Low/Low: 27.8% Int: 12.5% High: 15.3% Very High: 25.7% Missing: 18.8%	NR	RA or RARS: 15.6% RCMD: 13.7% Low/Int: 28.9% High risk: 39.1% RAEB-1: 17.6% RAEB-2: 21.5% CMML: 9% MDS-U: 23% T-MDS: 35.9%	Median (IQR), µg/L: 1131 (521–2246)
Patnaik, 2010 [29] USA	Retrospective cohort NR/unclear 88	Median (range): 74 (28–89) years	68.2%	NR	Transfusion need at diagnosis: 69%	NR	NR	MDS with isolated del(5q): 100%	At diagnosis: median (range), µg/L: 330 * (8–3599)
Prem, 2020 [30] Canada	Retrospective cohort Cancer center 125	≤65 years: 69.6% >65 years: 30.4%	66.1%	NR	44.8%	IPSS score: Low: 5.7% Int-1: 16.9% Int-2: 56.5% High: 21% Missing: *n* = 2 IPSS-R score: Very Low/Low: 12.2% Intermediate: 20.3% High: 33.3% Very High: 34.2% Missing: *n* = 2	NR	MDS subtype: RA/RCMD/Hypoplastic MDS: 36% RAEB-1: 20.8% RAEB-2: 43.2%	>1000 ng/mL: 52.5% of patients ≤1000 ng/mL: 47.5% of patients
Risum, 2016 [31] Denmark	Prospective cohort hematologic center at hospital 60	Median (range), years: 75.5 (46–94)	63.3%	Median (range), months: 16.5 (0.5–186.5)	35%	At diagnosis: (*n*) IPSS: (out of 56) Low: 24 Int-1: 24 Int-2: 5 High: 3 IPSS-R: (out of 56) Very Low: 12 Low: 29 Int: 7 High: 4 Very High: 4 At time of TE: (*n*) IPSS: (out of 57) Low: 29 Int-1: 16 Int-2: 5 High: 7 IPSS-R: (out of 57) Very Low: 21 Low: 18 Int: 6 High: 5 Very High: 7	NR	At time of TE: (*n*) RA: 2 RARS: 16 RCMD: 19 RAEB-1: 4 RAEB-2: 4 MDS del(5q): 5 AML (progressed from MDS): 3 CMML: 6 MDS/MPN: 1	Unclear timepoint: Transfusion dependent (*n* = 21), median (range), µg/L: 1035 (249–30,195); Transfusion independent (*n* = 39), median (range), µg/L: 390 (6–1866)
Senturk-Yikilmaz, 2019 [32] Turkey	Retrospective cohort Hospital 62	Mean (SD), years: 67.7 (12.3)	67.7%	NR	NR	NR	NR	MDS subtype: SF ≥ 400 ng/mL: RA: 9.7% RARS: 1.6% RCMD: 14.5% RCMD-RS: 1.6% RAEB-1/-2: 19.4% SF < 400 ng/mL: RA: 22.6% RARS: 1.6% RCMD: 19.4% RCMD-RS: 3.2% RAEB-1/-2: 6.5%	Median (range), ng/mL: 358.5 (29.8–2000)
Sperr, 2010 [33] Austria	Retrospective cohort Outpatient or clinic hematologic center 419	Median (IQR), years: 71 (24–91)	54.4%	NR	NR	Low: 135 (32.2%) Int-1: 158 (37.7%) Int-2: 79 (18.9%) High: 47 (11.2%)	RA: 128 (30.5%) RARS: 94 (23.4%) RAEB: 109 (26.0%) RAEB-t: 63 (15.0%) CMML: 25 (6.0%)	NR	NR
Wong, 2018 [35] Canada	Retrospective cohort Hospital 182	ICT patients, median (range), years: 67 (32–87); Non-ICT patients, median (range), years: 74 (39–93)	ICT: 60.3% Non-ICT: 57.1%	NR	NR	ICT: Low: 47.6% Int-1: 42.9% ≤Int-1: 9.5% Non-ICT: Low: 38.7% Int-1: 58.0% ≤Int-1: 3.4%	FAB or WHO, depending on era ICT: RA: 20.6% RARS, RARS-t: 44.4% RCMD, RCMD-RS: 23.8% del(5q): 4.8% RAEB-1: 3.2% MDS-U/MDS/MPN-U: 3.2% Non-ICT: RA: 20.2% RARS, RARS-t: 26.1% RCMD, RCMD-RS: 31.1% del(5q): 5.0% RAEB-1: 10.1% MDS-U/MDS/ MPN-U: 9.4%	FAB or WHO, depending on era ICT: RA: 20.6% RARS, RARS-t: 44.4% RCMD, RCMD-RS: 23.8% del(5q): 4.8% RAEB-1: 3.2% MDS-U/MDS/MPN-U: 3.2% Non-ICT: RA: 20.2% RARS, RARS-t: 26.1% RCMD, RCMD-RS: 31.1% del(5q): 5.0% RAEB-1: 10.1% MDS-U/MDS/MPN-U: 9.4%	Median (range), ng/mL: ICT: 687 (49–6447); Non-ICT: 260 (31–7783)
Osanai, 2018 [27] Japan	Retrospective cohort NR/unclear 98	Median (range), years: 71 (20–91)	60.2%	NR	NR	NR	NR	NR	NR

* Except when indicated otherwise. Abbreviations: CMML, chronic myelomonocytic leukemia; del(5q), deletion of part of long arm of human chromosome 5; FAB, French–American–British; HSCT, hematopoietic stem cell transplant; ICT, iron chelation therapy; Int, Intermediate; IPSS, International Prognostic Scoring System; IPSS-R, Revised International Prognostic Scoring System; IQR, interquartile range; MDS, myelodysplastic syndromes; MDS-F, MDS with fibrosis; MDS-MLD, MDS with multilineage dysplasia; MDS-RS-MLD, MDS with ring sideroblasts and multilineage dysplasia; MDS-RS-SLD, MDS with ring sideroblasts and single lineage dysplasia; MDS-SLD, MDS with single lineage dysplasia; MDS-U, myelodysplastic syndromes, unclassifiable; MPN, myeloproliferative neoplasm; MPN-RS-T, MPN with ring sideroblasts and thrombocytosis; MPN-U, MPN, unclassifiable; MU, myelodysplasia unspecified; NA, not applicable; NR, not reported; RA, refractory anemia; RAEB, refractory anemia with excess blasts; RAEB-t, refractory anemia with excess blasts in transformation; RARS, refractory anemia with ring sideroblasts; RARS-t, refractory anemia with ring sideroblasts with thrombocytosis; RBC, red blood cell; RCMD, refractory cytopenia with multilineage dysplasia; RCMD-RS, refractory cytopenia with multilineage dysplasia and ring sideroblasts; RCUD, refractory cytopenia with unilineage dysplasia; SD, standard deviation; SF, serum ferritin; TE, transient elastography; T-MDS, treatment-related myelodysplastic syndromes; WHO, World Health Organization.

## Data Availability

The data that support the findings of this study are available in the Supplementary Material of this article and from the corresponding author upon reasonable request.

## Supplementary Materials

The following are available online at https://www.mdpi.com/article/10.3390/jcm11030895/s1, Table S1: MEDLINE (via Ovid) Search Strategy for MDS Population, Table S2: Embase (via Ovid) Search Strategy for MDS Population, Table S3: MEDLINE (via Ovid) Search Strategy for MDS Population, Table S4: SF and Survival Outcomes in MDS Studies, Table S5: SF and PD and Relapse-Related Outcomes in MDS Studies, Table S6: Serum Ferritin and Other Outcomes in MDS Studies.
