# Supplementary material for: A Systematic Literature Review of the Relationship between Serum Ferritin and Outcomes in Myelodysplastic Syndromes
Source: J Clin Med. 2022 Feb 8;11(3):895. doi: 10.3390/jcm11030895 (PMC8836890; doi:10.3390/jcm11030895)
Supplement: Supplementary file 1 [file jcm-11-00895-s001.zip › jcm-1577829-supplementary.pdf]

SUPPLEMENTARY MATERIALS

Table S1. MEDLINE (via Ovid) Search Strategy for MDS Population.

Search Number	Search String
1.	exp Myelodysplastic Syndromes/ or myelodysplas*.tw. or mds.tw.
2.	exp Iron-Binding Proteins/ or serum ferritin.mp.
3.	1 and 2
4.	(animals not humans).sh.
5.	(case report or case series or woman or man or child or adolescent or female or male or boy or girl or infant).ti.
6.	case reports/ or case report$.jw.
7.	(Ephemera or Introductory Journal Article or News or Newspaper Article or Editorial or Comment or Overall or Letter or Congress).pt. or In Vitro Techniques/ or (commentary or editorial or comment or letter or congress or mice or rat or mouse or animal or murine).ti.
8	review.pt. not (systematic or meta$).mp.
9.	3 not (4 or 5 or 6 or 7 or 8)
10.	(pediatric$ or paediatric$ or preterm$ or newborn$ or child$ or infant$ or infancy or neonat$ or preschool$ or young$ or early years or adolescen$ or teenage$ or preteen$ or youth or girl$ or boy$ or student$ or juvenile$ or minor or minors or baby or babies).ti.
11.	(pediatric$ or paediatric$ or preterm$ or newborn$ or child$ or infant$ or infancy or neonat$ or preschool$ or young$ or early years or adolescen$ or teenage$ or preteen$ or youth or girl$ or boy$ or student$ or juvenile$ or minor or minors or baby or babies).ti. and (exp adult/ or adult$.ti.)
12.	10 not 11
13.	9 not 12
14.	limit 9 to english language
15.	limit 10 to yr="2009 -Current"

Abbreviation: MDS, myelodysplastic syndromes.

Table S2. Embase (via Ovid) Search Strategy for MDS Population.

Search Number	Search String
1.	exp Myelodysplastic Syndrome/ or myelodysplas$.tw. or mds.tw.
2.	exp ferritin blood level/ or serum ferritin.mp.
3.	1 and 2
4.	limit 3 to (article or article in press)
5.	(animal not human).sh.
6.	(case report or case series or woman or man or child or adolescent or female or male or boy or girl or infant).ti.
7.	case study/ or case report$.jx. or case report$.jw.
8	(editorial or note or letter).pt. or in vitro Techniques/ or in vitro study/ or (commentary or editorial or comment or letter or mice or rat or mouse or animal or murine).ti.
9.	review.pt. not (systematic or meta$).mp.
10.	4 not (5 or 6 or 7 or 8 or 9)
11.	(pediatric$ or paediatric$ or preterm$ or newborn$ or child$ or infant$ or infancy or neonat$ or preschool$ or young$ or early years or adolescen$ or teenage$ or preteen$ or youth or girl$ or boy$ or student$ or juvenile$ or minor or minors or baby or babies).ti.
12.	(pediatric$ or paediatric$ or preterm$ or newborn$ or child$ or infant$ or infancy or neonat$ or preschool$ or young$ or early years or adolescen$ or teenage$ or preteen$ or youth or girl$ or boy$ or student$ or juvenile$ or minor or minors or baby or babies).ti. and (exp adult/ or adult$.ti.)
13.	11 not 12
14.	10 not 13
15.	limit 14 to english language
16.	Limit 11 to yr="2009 -Current"

Abbreviation: MDS, myelodysplastic syndromes.

Table S3. MEDLINE (via Ovid) Search Strategy for MDS Population.

**Author and Year of Publication**	**Summary Study Participation**	**Study Attrition Summary**	**PF Measurement Summary**	**Outcome Measurement Summary**	**Study Confounding Summary**	**Statistical Analysis and Presentation Summary**
	The study sample represents the population of interest on key characteristics, sufficient to limit potential bias of the observed relationship between PF and outcome	Loss to follow-up (from baseline sample to study population analyzed) is not associated with key characteristics (i.e., the study data adequately represent the sample) sufficient to limit potential bias to the observed relationship between PF and outcome	*PF* is adequately measured in study participants to sufficiently limit potential bias	*Outcome of interest* is adequately measured in study participants to sufficiently limit potential bias	Important potential confounders are appropriately accounted for, limiting potential bias with respect to the relationship between *PF* and *outcome*	The statistical analysis is appropriate for the design of the study, limiting potential for presentation of invalid or spurious results
Irwin, 2011 [20]	Low Risk	Low Risk	Low Risk	Low Risk	Low Risk	Low Risk
Park, 2011 [28]	Low Risk	Low Risk	Low Risk	Low Risk	Low Risk	Low Risk

Waszczuk-Gajda, 2016 [34]	Moderate Risk	Low Risk	Low Risk	Low Risk	Low Risk	Low Risk
Cakar, 2013 [15]	Moderate Risk	Low Risk	Low Risk	Low Risk	Low Risk	Low Risk
Cermak, 2009 [16]	Moderate Risk	Low Risk	Low Risk	Low Risk	Low Risk	Low Risk
Cremers, 2019 [17]	Low Risk	Low Risk	Low Risk	Low Risk	Low Risk	Low Risk
Diamantopoulos, 2019 [18]	Low Risk	Low Risk	Low Risk	Low Risk	Low Risk	Low Risk
Escudero-Vilaplana, 2015 [19]	Low Risk	Low Risk	Low Risk	Low Risk	Moderate Risk	Low Risk
Kadlckova, 2017 [21]	Moderate Risk	Low Risk	Low Risk	Low Risk	Moderate Risk	Low Risk
Kawabata, 2019 [22]	Low Risk	Low Risk	Low Risk	Low Risk	Low Risk	Low Risk
Kikuchi, 2012 [23]	Low Risk	Low Risk	Low Risk	Low Risk	Low Risk	Low Risk
Li, 2013 [24]	Low Risk	Low Risk	Low Risk	Low Risk	Low Risk	Low Risk
Lucijanic, 2016 [25]	Moderate Risk	Low Risk	Low Risk	Low Risk	Low Risk	Low Risk
Oran, 2014 [26]	Low Risk	Low Risk	Low Risk	Low Risk	Low Risk	Low Risk
Patnaik, 2010 [29]	Low Risk	Low Risk	Low Risk	Low Risk	Low Risk	Low Risk
Prem, 2020 [30]	Low Risk	Low Risk	Low Risk	Low Risk	Moderate Risk	Low Risk
Risum, 2016 [31]	Low Risk	Low Risk	Low Risk	Low Risk	Low Risk	Low Risk
Senturk-Yikilmaz, 2019 [32]	Low Risk	Low Risk	Low Risk	Low Risk	Low Risk	Low Risk

Sperr, 2010 [33]	Low Risk	Low Risk	Low Risk	Low Risk	Low Risk	Low Risk
Wong, 2018 [35]	Low Risk	Low Risk	Low Risk	Low Risk	Low Risk	Low Risk

Abbreviation: PF, prognostic factor.

Table S4. SF and Survival Outcomes in MDS Studies.

**Study Author, Year**<br>**Country**<br>**Study Design**	**Population (*n*)**	**Univariate or Multivariate and Type of Statistical Analysis Performed**	**Model Variables**	**Time Point at Which SF Evaluated**	**Continuous or Categorical, and Categories**	**Outcome**	**Effect**
Park, 2011 [28]<br>France<br>Retrospective cohort	Overall<br>318	Univariate<br>Wilcoxon's test	NR	Diagnosis<br>(Baseline)	Categorical<br>SF >300 ng/mL<br>(*n* = 153)	OS	*p* = 0.98
	Overall<br>318	Univariate<br>Wilcoxon's test	NR	Diagnosis<br>(Baseline)	Categorical<br>SF >1000<br>ng/mL (*n* = 32)	OS	*p* = 0.67
Waszczuk-Gajda, 2016 [34]<br>Poland<br>Retrospective cohort	Overall<br>190	Univariate<br>Cox proportional hazards	NR	Diagnosis<br>(Baseline)	Categorical<br>SF >1000<br>ng/mL (*n* = 20)	Worsened survival	HR: 2.94<br>*p* = 0.0023
Cermak, 2009 [16]<br>Czech Republic<br>Retrospective cohort	Overall<br>137	Univariate<br>Log rank test	NR	Most recent available	Categorical<br>SF >2000 µg/L<br>(*n* = 69)	OS	*p* = 0.135
	Subgroup: Non-transplanted patients	Univariate<br>Log rank test	NR	Most recent available	Categorical<br>SF >2000 µg/L<br>(*n* = 69)	OS	*p* = 0.049

Study Author, Year<br>Country<br>Study Design	Population (*n*)	Univariate or Multivariate and Type of Statistical Analysis Performed	Model Variables	Time Point at Which SF Evaluated	Continuous or Categorical, and Categories	Outcome	Effect
	107						
	Overall<br>137	Multivariate<br>Cox regression analysis	WHO classification, IPSS subgroup, age, SF levels, progressive disease, RBC units/month	Most recent available	Categorical<br>SF >2000 µg/L (*n* = 69)	OS	$p = 0.007$
Cremers, 2019 [17]<br>European multi-country<br>Prospective cohort	Overall<br>222	Multivariate<br>Cox proportional hazards	RBC transfusions, CRP levels, SF levels (continuous in units of 1000 ng/mL) and comorbidities, WHO classification, age at HSCT, donor type, sex-match, and intensity of conditioning regimen	Baseline	Continuous (units of 1000 ng/mL)	OS	HR (95% CI): 1.2 (1–1.4); $p = 0.05$
	Overall<br>222	Multivariate<br>Cox proportional hazards	RBC transfusions, CRP levels, SF levels (continuous in units of 1000 ng/mL) and comorbidities, WHO classification, age at HSCT, donor type, sex-match,	Baseline	Continuous (units of 1000 ng/mL)	NRM	HR (95% CI): 1.1 (0.8–1.4); $p = 0.6$

Study Author, Year<br>Country<br>Study Design	Population (*n*)	Univariate or Multivariate and Type of Statistical Analysis Performed	Model Variables	Time Point at Which SF Evaluated	Continuous or Categorical, and Categories	Outcome	Effect
			and intensity of conditioning regimen				
Diamantopoulos, 2019 [18]<br>Greece<br>Retrospective cohort	Overall<br>88	Univariate<br>Log rank test	NR	Diagnosis<br>(Baseline)	Categorical<br>SF >400 ng/mL<br>(*n* = NR)	OS	*p* = 0.003
	Overall<br>88	Multivariate<br>NR	CMML-1/2, Hb <10 g/dL, SF >400 ng/mL, PLT <100 x 10^9/L and circulating blasts	Diagnosis<br>(Baseline)	Categorical<br>SF >400 ng/mL<br>(*n* = NR)	OS	HR (95% CI): 2.84 (1.16–6.94); *p* = 0.022
Kadlckova, 2017 [21]<br>Czech Republic<br>Prospective cohort	Overall<br>73	Multivariate<br>Cox proportional hazards	NR	Diagnosis<br>(Baseline)	Continuous	OS	HR: 1.2<br>*p*<0.001
Kawabata, 2019 [22]<br>Japan<br>Prospective cohort	Overall<br>107<br>Low SF group: 56	Multivariate<br>Cox proportional hazards	Age, sex, Hb level, neutrophil count, platelet count, BM blast percentage, and cytogenetic	Diagnosis<br>(Baseline)	Categorical<br>SF ≥210 ng/mL<br>(*n* = 51)	OS	HR (95% CI): 2.14 (1.02–4.50);

**Study Author, Year Country Study Design**	**Population (*n*)**	**Univariate or Multivariate and Type of Statistical Analysis Performed**	**Model Variables**	**Time Point at Which SF Evaluated**	**Continuous or Categorical, and Categories**	**Outcome**	**Effect**
	High SF group: 51		score (based on the IPSS-R) in addition to SF level				$p = 0.044$
Kikuchi, 2012 [23] Japan Retrospective cohort	Overall 47 Low SF group: 37 High SF group: 10	Univariate Logistic regression	NA; univariate	Diagnosis (Baseline)	Categorical SF ≥500 ng/mL (*n* = 10)	OS	HR (95% CI): 10.7 (2.375–48.23); $p = 0.002$
	Overall 47 Low SF group: 37 High SF group: 10	Multivariate Cox proportional hazards	Cytogenetic risk (intermediate and poor), IPSS subgroup (Int-2 and High), and SF at diagnosis ≥500 ng/mL	Diagnosis (Baseline)	Categorical SF ≥500 ng/mL (*n* = 10)	OS	HR (95% CI): 1.9 (1.033–3.497); $p = 0.039$
	Overall 47 Low SF group:	Multivariate Cox proportional hazards	NR	Diagnosis (Baseline)	Categorical SF ≥300 ng/mL (*n* = NR)	OS	HR (95% CI): 3.437

Study Author, Year<br>Country<br>Study Design	Population (n)	Univariate or Multivariate and Type of Statistical Analysis Performed	Model Variables	Time Point at Which SF Evaluated	Continuous or Categorical, and Categories	Outcome	Effect
	37<br>High SF group: 10						(1.140–10.36);<br>$p = 0.028$
Li, 2013 [24]<br>China<br>Prospective cohort	Overall<br>191	Univariate<br>Log rank test	NA; univariate	Baseline	Categorical<br>SF ≥500 µg/L<br>($n = 74$)	OS	$p < 0.001$
	Overall<br>191	Multivariate<br>Cox proportional hazards	ECOG PS score, ANC, SF, percentage of BM blasts, and poor karyotype	Baseline	Categorical<br>SF ≥500 µg/L<br>($n = 74$)	OS	RR (95% CI): 3.53 (1.9–6.6);<br>$p < 0.001$
	Subgroup: Patients with prior RBC transfusion<br>83	Multivariate<br>Cox proportional hazards	ECOG PS score, ANC, SF, percentage of BM blasts, and poor karyotype	Baseline	Categorical<br>SF ≥500 µg/L<br>($n = 74$)	OS	RR (95% CI): 2.876 (1.612–5.131);<br>$p < 0.001$

**Study Author, Year**<br>**Country**<br>**Study Design**	**Population (*n*)**	**Univariate or Multivariate and Type of Statistical Analysis Performed**	**Model Variables**	**Time Point at Which SF Evaluated**	**Continuous or Categorical, and Categories**	**Outcome**	**Effect**
	Subgroup: Patients without prior RBC transfusion<br>108	Multivariate<br>Cox proportional hazards	ECOG PS score, ANC, SF, percentage of BM blasts, and poor karyotype	Baseline	Categorical<br>SF ≥500 µg/L<br>(n = 74)	OS	RR (95% CI): 3.363 (1.509–7.495);<br>p = 0.003
Oran, 2014 [26]<br>US<br>Retrospective cohort	Overall<br>256	Univariate<br>Log rank test	NR	All outcomes were measured from the time of stem cell infusion	Categorical<br>SF >1130 µg/L<br>(n = NR)	TRM	HR: 2.0<br>p = 0.009
	Overall<br>256	Univariate<br>Log rank test	NR	All outcomes were measured from the time of stem cell infusion	Categorical<br>SF >1130 µg/L<br>(n = NR)	OS	HR: 2.0<br>p = 0.001
	Overall<br>256	Univariate<br>Log rank test	NR	All outcomes were measured from the	Categorical	TRM	HR: 1.2<br>p = 0.6

Study Author, Year<br>Country<br>Study Design	Population (*n*)	Univariate or Multivariate and Type of Statistical Analysis Performed	Model Variables	Time Point at Which SF Evaluated	Continuous or Categorical, and Categories	Outcome	Effect
				time of stem cell infusion	SF missing *versus* ≤1130 µg/L (*n* = NR)		
	Overall<br>256	Univariate<br>Log rank test	NR	All outcomes were measured from the time of stem cell infusion	Categorical<br>SF missing *versus* ≤1130 µg/L (*n* = NR)	OS	HR: 1.7<br>$p = 0.02$
	Overall<br>256	Multivariate<br>Cox proportional hazards or Fine and Gray method	Age, SF level at HSCT, donor type, conditioning intensity, and transplantation year	All outcomes were measured from the time of stem cell infusion	Categorical<br>SF >1150 µg/L (*n* = NR)	TRM	HR: 1.7<br>$p = 0.06$
	Overall<br>256	Multivariate<br>Cox proportional hazards or Fine and Gray method	Age, histological subtype, T-MDS, MK, SF, and BM blast count at HSCT, donor type, and transplantation year	All outcomes were measured from the time of stem cell infusion	Categorical<br>SF >1150 µg/L (*n* = NR)	OS	HR: 2.2<br>$p < 0.001$

Study Author, Year<br>Country<br>Study Design	Population (*n*)	Univariate or Multivariate and Type of Statistical Analysis Performed	Model Variables	Time Point at Which SF Evaluated	Continuous or Categorical, and Categories	Outcome	Effect
	Overall<br>256	Multivariate<br>Cox proportional hazards or Fine and Gray method	Age, SF level at HSCT, donor type, conditioning intensity, and transplantation year	All outcomes were measured from the time of stem cell infusion	Categorical<br>SF missing *versus* ≤1150 µg/L (*n* = NR)	TRM	HR: 0.7<br>*p* = 0.4
	Overall<br>256	Multivariate<br>Cox proportional hazards or Fine and Gray method	Age, histological subtype, T-MDS, MK, SF, and BM blast count at HSCT, donor type, and transplantation year	All outcomes were measured from the time of stem cell infusion	Categorical<br>SF missing *versus* ≤1150 µg/L (*n* = NR)	OS	HR: 1.1<br>*p* = 0.2
Patnaik, 2010 [29]<br>USA<br>Retrospective cohort	Overall<br>88	Univariate<br>Chi-squared test	NR	Diagnosis (Baseline)	Continuous	OS	*p* = 0.21
Prem, 2020 [30]<br>Canada<br>Retrospective cohort	Overall<br>125	Univariate<br>Log rank test	NR	Pretransplant SF levels were assessed within 30 days prior to admission for transplant	Categorical<br>SF ≤1000 (*n* = 57) *versus* >1000 ng/mL (*n* = 63)	OS	HR (95% CI): 1.85 (1.17–2.94);<br>*p* = 0.0086

**Study Author, Year**<br>**Country**<br>**Study Design**	**Population (*n*)**	**Univariate or Multivariate and Type of Statistical Analysis Performed**	**Model Variables**	**Time Point at Which SF Evaluated**	**Continuous or Categorical, and Categories**	**Outcome**	**Effect**
	Overall<br>125	Multivariate<br>Cox proportional hazards	NR	Pretransplant SF levels were assessed within 30 days prior to admission for transplant	Categorical<br>SF ≤1000 (*n* = 57) *versus* >1000 ng/mL (*n* = 63)	OS	HR (95% CI): 1.729 (1.085–2.754);<br>$p = 0.0212$
Senturk-Yikilmaz, 2019 [32]<br>Turkey<br>Retrospective cohort	Overall<br>62	Univariate<br>Cox proportional hazards	NR	Diagnosis (Baseline)	Categorical<br>SF ≥400 ng/mL (*n* = 29)	OS	$p = 0.001$
Sperr, 2010 [33]<br>Austria<br>Retrospective cohort	Overall<br>419	Multivariate<br>Cox proportional hazards	Age, LDH levels, SF, FAB subgroup, number of cytopenias, and karyotype (per IPSS criteria)	Baseline	Continuous	OS	HR: 2.2<br>$p < 0.01$
	Subgroup: Low or Int-1 patients<br>293	Multivariate<br>Cox proportional hazards	Age, LDH levels, SF, FAB subgroup, number of cytopenias, and karyotype (per IPSS criteria)	Baseline	Continuous	OS	HR: 2.5<br>$p < 0.01$

Study Author, Year<br>Country<br>Study Design	Population (*n*)	Univariate or Multivariate and Type of Statistical Analysis Performed	Model Variables	Time Point at Which SF Evaluated	Continuous or Categorical, and Categories	Outcome	Effect
	Subgroup: Int-2 or high patients<br>126	Multivariate<br>Cox proportional hazards	Age, LDH levels, SF, FAB subgroup, number of cytopenias, and karyotype (per IPSS criteria)	Baseline	Continuous	OS	HR: 1.8<br>*p* = NS
Wong, 2018 [35]<br>Canada<br>Retrospective cohort	Overall<br>182	Multivariate<br>Cox proportional hazards	Age at MDS diagnosis, IPSS score, SF, MDS diagnosis, ICT	Baseline	Continuous	OS	HR (95% CI): 1.0 (1–1); NR<br>SD: 0<br>*p* = 0.7
Osanai, 2018 [27]<br>Japan<br>Retrospective cohort	Overall<br>98	Multivariate<br>Cox proportional hazards	NR	Diagnosis (baseline)	Categorical<br>SF >500 ng/mL<br>(*n* = NR)	OS	HR (95% CI): 3.04 (1.58–5.70);<br>*p* = 0.0012

Abbreviations: ANC, absolute neutrophil count; BM, bone marrow; CI, confidence interval; CMML, chronic myelomonocytic leukemia; CRP, C-reactive protein; ECOG PS, Eastern Cooperative Oncology Group performance status; Hb, hemoglobin; HR, hazard ratio; HSCT, hematopoietic stem cell transplant; ICT, iron chelation therapy; IPSS, International Prognostic Scoring System; IPSS-R, revised International Prognostic Scoring System; LDH, lactate dehydrogenase; MDS, myelodysplastic syndromes; MK,

monosomal karyotype; NA, not applicable; NRM, non-relapse mortality; NR, not reported; NS, not significant; OS, overall survival; PLT, platelet; RBC, red blood cell; RR, relative risk; SF, serum ferritin; TRM, treatment-related mortality; WHO, World Health Organization.

Table S5. SF and PD and Relapse-Related Outcomes in MDS Studies.

**Study Author, Year<br>Country<br>Study Design**	**Population (*n*)**	**Univariate or Multivariate and Type of Statistical Analysis Performed**	**Model Variables**	**Time point at Which SF Evaluated**	**Continuous or Categorical, and Categories**	**Outcome**	**Effect**
Park, 2011 [28]<br>France<br>Retrospective cohort	Overall<br>318	Univariate<br>Wilcoxon's test	NR	Diagnosis<br>(Baseline)	Categorical<br>SF >300 ng/mL (*n* = 153)	Transformation to AML	$p = 0.94$
	Overall<br>318	Univariate<br>Wilcoxon's test	NR	Diagnosis<br>(Baseline)	Categorical<br>SF >1000 ng/mL (*n* = 32)	Transformation to AML	$p = 0.47$
Waszczuk-Gajda, 2016 [34]<br>Poland<br>Retrospective cohort	Overall<br>190	Univariate<br>Chi-squared test	NR	Diagnosis<br>(Baseline)	Categorical<br>SF >1000 ng/mL (*n* = 20)	Transformation to AML	$p > 0.05$
	Overall<br>190	Univariate<br>Chi-squared test	NR	Diagnosis<br>(Baseline)	Categorical<br>SF >1000 ng/mL (*n* = 20)	Time to transformation to AML	$p = 0.35$

Study Author, Year<br>Country<br>Study Design	Population (*n*)	Univariate or Multivariate and Type of Statistical Analysis Performed	Model Variables	Time point at Which SF Evaluated	Continuous or Categorical, and Categories	Outcome	Effect
Cremers, 2019 [17]<br>European multi-country<br>Prospective cohort	Overall<br>222	Multivariate<br>Cox proportional hazards	One by one the other variables of specific interest in the current study: RBC transfusions, CRP levels, SF levels (continuous in units of 1000 ng/mL) and comorbidities, WHO classification, age at HSCT, donor type, sex-match, and intensity of conditioning regimen	Baseline	Continuous (units of 1000 ng/mL)	Relapse incidence	HR (95% CI): 1.3 (1.01–1.6); $p = 0.04$
	Overall<br>222	Multivariate<br>Cox proportional hazards	One by one the other variables of specific interest in the current study: RBC transfusions, CRP levels, SF levels (continuous in units of 1000 ng/mL) and comorbidities, WHO-classification, age at HSCT, donor type, sex-match, and intensity of conditioning regimen	Baseline	Continuous (units of 1000 ng/mL)	RFS	HR (95% CI): 1.2 (0.98–1.4); $p = 0.08$

**Study Author, Year**<br>**Country**<br>**Study Design**	**Population (*n*)**	**Univariate or Multivariate and Type of Statistical Analysis Performed**	**Model Variables**	**Time point at Which SF Evaluated**	**Continuous or Categorical, and Categories**	**Outcome**	**Effect**
Kadlckova, 2017 [21]<br>Czech Republic<br>Prospective cohort	Overall<br>73	Multivariate<br>Cox proportional hazards	NR	Diagnosis (Baseline)	Continuous	EFS	HR: 1.14<br>$p = 0.001$
Oran, 2014 [26]<br>US<br>Retrospective cohort	Overall<br>256	Univariate<br>Log rank test	NR	All outcomes were measured from the time of stem cell infusion	Categorical<br>SF >1130 µg/L (n = NR)	Relapse incidence	HR: 1.0<br>$p = 0.8$
	Overall<br>256	Univariate<br>Log rank test	NR	All outcomes were measured from the time of stem cell infusion	Categorical<br>SF >1130 µg/L (*n* = NR)	EFS	HR: 1.6<br>$p = 0.01$
	Overall<br>256	Univariate<br>Log rank test	NR	All outcomes were measured from the time of stem cell infusion	Categorical<br>SF missing *versus* ≤1130 µg/L (*n* = NR)	Relapse incidence	HR: 1.7<br>$p = 0.06$

Study Author, Year<br>Country<br>Study Design	Population (*n*)	Univariate or Multivariate and Type of Statistical Analysis Performed	Model Variables	Time point at Which SF Evaluated	Continuous or Categorical, and Categories	Outcome	Effect
	Overall<br>256	Univariate<br>Log rank test	NR	All outcomes were measured from the time of stem cell infusion	Categorical<br>SF missing *versus* ≤1130 μg/L (*n* = NR)	EFS	HR: 1.5<br>*p* = 0.05
	Overall<br>256	Multivariate<br>Cox proportional hazards or Fine and Gray method	Age, histological subtype, T-MDS, MK, SF levels, BM blast count at HSCT, and transplantation year	All outcomes were measured from the time of stem cell infusion	Categorical<br>SF >1150 μg/L (*n* = NR)	EFS	HR: 1.8<br>*p* = 0.002
	Overall<br>256	Multivariate<br>Cox proportional hazards or Fine and Gray method	Age, histological subtype, T-MDS, MK, SF, BM blast count at HSCT and transplantation year	All outcomes were measured from the time of stem cell infusion	Categorical<br>SF missing *versus* ≤1150 μg/L (n = NR)	EFS	HR: 1.0<br>*p* = 0.9
Prem, 2020 [30]<br>Canada<br>Retrospective cohort	Overall<br>125	Univariate<br>Log rank test	NR	Pretransplant ferritin levels were assessed within 30 days prior to	Categorical<br>SF ≤1000 *versus* >1000 ng/mL	RFS	HR (95% CI): 1.931 (1.239–30.10);

**Study Author, Year Country Study Design**	**Population (*n*)**	**Univariate or Multivariate and Type of Statistical Analysis Performed**	**Model Variables**	**Time point at Which SF Evaluated**	**Continuous or Categorical, and Categories**	**Outcome**	**Effect**
				admission for transplant			p = 0.0037
	Overall 125	Multivariate Cox proportional hazards	NR	Pretransplant SF levels were assessed within 30 days prior to admission for transplant	Categorical SF ≤1000 *versus* >1000 ng/mL	RFS	HR (95% CI): 1.799 (1.147–2.823); p = 0.0106
Kikuchi, 2012 [23] Japan Retrospective cohort	Overall: 47 Low SF group: 37 High SF group: 10	Univariate Logistic regression	NR	Diagnosis (Baseline)	Categorical SF ≥500 ng/mL (n = 10)	LFS	HR (95% CI): 21.16 (2.062–217.1); p = 0.01
	Overall:	Univariate Logistic regression	NR	Diagnosis (Baseline)	Categorical	LFS	HR (95% CI): 4.752

Study Author, Year<br>Country<br>Study Design	Population (n)	Univariate or Multivariate and Type of Statistical Analysis Performed	Model Variables	Time point at Which SF Evaluated	Continuous or Categorical, and Categories	Outcome	Effect
	47<br>Low SF group: 37<br>High SF group: 10				SF ≥300 ng/mL (n = NR)		(0.852–26.51); $p = 0.076$
Sperr, 2010 [33]<br>Austria<br>Retrospective cohort	Overall: 419	Multivariate Cox proportional hazards	Age, LDH levels, SF levels, FAB subgroup, number of cytopenias, and karyotype (per IPSS criteria)	Baseline	Continuous	EFS	HR: 2.0<br>$p < 0.01$
	Subgroup: Low or Int-1 patients<br>293	Multivariate Cox proportional hazards	Age, LDH levels, SF levels, FAB subgroup, number of cytopenias, and karyotype (per IPSS criteria)	Baseline	Continuous	EFS	HR: 2.9<br>$p < 0.01$
	Subgroup: Int-2 or High patients<br>126	Multivariate Cox proportional hazards	Age, LDH levels, SF levels, FAB subgroup, number of cytopenias, and karyotype (per IPSS criteria)	Baseline	Continuous	EFS	HR: 1.2<br>p = NS

Abbreviations: AML, acute myeloid leukemia; BM, bone marrow; CI, confidence interval; CRP, C-reactive protein; EFS, event-free survival; FAB, French–American–British; HR, hazard ratio; HSCT, hematopoietic stem cell transplant; IPSS, International Prognostic Scoring System; LDH, lactate dehydrogenase; LFS, leukemia-free survival; MDS, myelodysplastic syndromes; MK, monosomal karyotype; NR, not reported; NS, not significant; RBC, red blood cell; RFS, relapse-free survival; SF, serum ferritin; T-MDS, treatment-related myelodysplastic syndromes; WHO, World Health Organization.

Table S6. Serum Ferritin and Other Outcomes in MDS Studies.

**Study Author, Year Country Study Design**	**Population (*n*)**	**Univariate or Multivariate and Type of Statistical Analysis Performed**	**Model Variables**	**When SF Evaluated**	**Continuous or Categorical, and Categories**	**Outcome**	**Effect**
Irwin, 2011 [20] New Zealand Retrospective cohort	Overall 70	Univariate Pearson product-moment correlation coefficient	NR	The SF level at diagnosis and the most recently performed SF level were recorded. As this was a retrospective analysis, the most recent SF of each patient was not always taken at the end of the study. For some patients, the most recent SF was performed after the bulk of their transfusions, while for others it was performed before a significant proportion of their transfusions had been administered	Continuous	Number of units of RBC transfused	Model coefficient: r = 0.290

**Study Author, Year**<br>**Country**<br>**Study Design**	**Population (*n*)**	**Univariate or Multivariate and Type of Statistical Analysis Performed**	**Model Variables**	**When SF Evaluated**	**Continuous or Categorical, and Categories**	**Outcome**	**Effect**
Cakar, 2013 [15]<br>Turkey<br>Retrospective cohort	Overall<br>35	Univariate<br>Mann–Whitney U test	NR	Diagnosis (Baseline)	Continuous	Treatment response	*p* = 0.004
Escudero-Vilaplana, 2015 [19]<br>Spain<br>Retrospective cohort	Overall<br>35	Multivariate<br>Pearson product-moment correlation coefficient or Spearman's rho test	NR	During treatment	Continuous	Medication adherence	Model coefficient: r = −0.288<br>*p* = 0.004
Lucijanic, 2016 [25]<br>Croatia<br>Prospective cohort	Overall<br>36	Univariate<br>Fisher exact test or Chi-squared test	NR	Parameters of iron metabolism were measured repeatedly during the follow-up period and the most recent follow-up data was used	Categorical<br>SF >1000 µg/L	Blood units received	Spearman Rho: 0.52<br>*p* = 0.04
Risum, 2016 [31]<br>Denmark<br>Prospective cohort	Overall<br>60	Univariate<br>Spearman's test	NR	Most patients had their SF measured within 1 day of the FibroScan, but some outpatients had	Categorical<br>High *versus* low SF*	Median liver stiffness measurement	*p* = 0.583

Study Author, Year<br>Country<br>Study Design	Population<br>(*n*)	Univariate or Multivariate and Type of Statistical Analysis Performed	Model Variables	When SF Evaluated	Continuous or Categorical, and Categories	Outcome	Effect
				their SF measured up to 19 days before or after the day of the FibroScan			

*High SF was >320 µg/L in men and >161 µg/L in women.

Abbreviations: NR, not reported; RBC, red blood cell; SF, serum ferritin.
